# Analysis of otoacoustic emissions in neonates at term and preterm

**DOI:** 10.5935/1808-8694.20130104

**Published:** 2015-10-08

**Authors:** Juliana Maria Soares Cavalcante, Myriam de Lima Isaac

**Affiliations:** a MSc - Medical School of Ribeirão Preto, University of São Paulo (FMRP-USP); PhD Student - FMRP-USP.; b PhD - FMRP-USP; Professor - FMRP-USP. Medical School of Ribeirão Preto, University of São Paulo.

**Keywords:** hearing, neonatal screening, newborn, premature, spontaneous otoacoustic emissions

## Abstract

The transient evoked otoacoustic emissions (TEOAEs) have been widely used in neonatal hearing screening.

**Objective:**

To compare the TEOAEs in newborns at term and preterm vis-à-vis the following variables: ear side, gender, frequency spectrum and gestational age.

**Method:**

By means of a cross-sectional cohort of 66 newborns up to the 28^th^ day of life (41 newborns at term and 25 premature babies), we recorded TEOAEs. All the individuals did not have risk indicators for hearing loss.

**Results:**

There was a signal/noise ratio improvement with frequency increase. No differences were observed between genders and between the ears, but there were differences among the children born at term and preterm in the frequency bands at 3 kHz and 4 kHz.

**Conclusion:**

The TEOAEs test is important for assessing the peripheral auditory system of newborns at term and preterm, making it possible to have responses regardless of gender and gestational age.

## INTRODUCTION

Auditory system integrity is important for an individual's communication and social interaction. Hearing enables the child to develop the verbal mode of communication, so it is essential to screen hearing at birth.

Many techniques are used for the evaluation of hearing sensitivity and, among them, we have otoacoustic emissions. There is an acoustic energy that can be measured in the external ear canal, spontaneously or evoked by sound stimuli. When evoked, these emissions are usually classified according to the generating stimulus: transient, frequency stimulus and distortion product^1^.

In his first publication on the subject, Kemp^2^ described the existence of a cochlear echo which could be measured in the external ear canal, originating from the biomechanics of the organ of Corti outer hair cells. These response components arise from a nonlinear cochlear mechanism in response to an acoustic stimulation.

In recent years, the recording of transient evoked otoacoustic emissions has been widely used in neonatal hearing screening of newborns without hearing risks, using low intensity stimuli, with a wide range of frequencies and in a short period of time^3^.

Chapchap^4^ reported that 98% of individuals with normal hearing and with hearing thresholds below 30 dB HL at any frequency, have transient evoked otoacoustic emissions (TEOAE). He emphasized that the method of TEOAE recording has the advantage of being stable, fast and non-invasive, enabling cochlear integrity monitoring. He noted that the newborn state of consciousness impacted the recording of responses and middle ear disorders interfered with stimulus transmission and response recording.

In a study assessing the peripheral hearing of 157 full term and preterm newborns, normal and small for gestational age, using transient otoacoustic emissions, Garcia et al.^5^ reported that premature infants failed this exam more than those born at full term. The prevalence of conductive hearing loss in the sample was 29 for every 1.000 ears and there was sensorineural hearing loss in 16 of 1.000 ears.

According to the organizations involved with pediatric hearing^6^, ^7^, 30% to 50% of children with significant hearing loss have no risk factors and, therefore, hearing screening is recommended for all newborns. Transient otoacoustic emissions are considered the main hearing screening procedure to be used in neonates without hearing risk.

In normative data measurement for transient otoacoustic emissions, some authors have observed a decrease in otoacoustic emissions with increasing age^8^.

Tognola et al.^9^ assessed the differences of otoacoustic emissions in full term and preterm newborns, from 34 preterm infants admitted to the NICU and compared with 333 term neonates without risk for hearing impairment, tested in the third day after birth. The authors found lower levels of premature infants on record and stated that the cochlea of the newborn is not fully developed until the 38^th^ week of gestational age and that after that age, the record becomes similar between full term and preterm newborns.

In a recent study^10^ the authors evaluated differences in the otoacoustic emissions full term and preterm newborns, and found higher response latency in preterm infants.

Duarte et al.^11^ analyzed the transient otoacoustic emissions in a program of newborn hearing screening, and found no differences in responses between infants born at full term and preterm.

Given the variety of findings found in the comparison of full term and preterm newborns, we felt the need to study transient otoacoustic emissions in full term and preterm newborns, without any hearing risk, in order to try to understand cochlear function maturation in this age group that has no risk of hearing.

### Objective

To compare the signal-to-noise ratio of transient evoked otoacoustic emissions in full term and preterm newborns, vis-à-vis the following variables: ear side, gender, frequency spectrum and gestational age.

## METHOD

The present investigation is a cross-sectional cohort study, developed after approval by the Ethics in Research Committee, under protocol 11447/2007.

### Series

To do this study, an evaluation was scheduled within the first 28 days of life, for the analysis of transient evoked otoacoustic emissions (TEOAE) of 66 infants in good health. All newborns were born and recruited at the University Hospital of the Medical School of Ribeirão Preto - University of São Paulo, and the test was scheduled at the time of hospital discharge in the child's release card. The mothers were informed about the study and all signed an informed consent form.

Two groups were formed: Group 1, with 41 healthy and full term newborns, 29 females and 12 males, with gestational age of 37 to 42 weeks; and Group 2, with 25 healthy preterm newborns, 13 females and 12 males, between 32-36 weeks of gestational age.

All children selected for this study had no risk indicators for hearing loss^7^ according to the criteria listed below:

• Family history of permanent hearing loss in childhood;
• NICU stay for more than five days, or the presence of any of the following risk factors, regardless of length of stay: mechanical ventilation, exposure to ototoxic drugs and hyperbilirubinemia serum level requiring exchange transfusion;
• Congenital infections such as cytomegalovirus, herpes, rubella, syphilis, and toxoplasmosis;
• Craniofacial anomalies, including disorders in the ear canal, ear marks, ear pits, and temporal bone abnormalities;
• Physical findings associated with other syndromes related to sensorineural hearing loss or permanent conductive hearing loss;
• Syndromes associated with progressive or late-onset hearing loss, such as neurofibromatosis, osteopetrosis, Usher syndrome, and other frequently identified syndromes including Waardenburg, Alport, Pendred, Jervell and Lange - Nielson;
• Neurodegenerative disorders, such as Hunter syndrome, or sensorimotor neuropathies, such as Friedreich's ataxia and Charcot-Marie-Tooth syndrome;
• Postnatal infections associated with sensorineural hearing loss, including bacterial and viral meningitis;
• Traumatic brain injury, especially temporal bone fractures that require hospitalization;
• Children who failed the newborn hearing screening performed by TEOAE.

### Method

The TEOAE was performed in a soundproof booth with the baby under natural sleep or quiet.

The equipment used was the Intelligent Hearing System SMART-EP. Newborns were evaluated in both ears. The stimulus was a click at the intensity of 80 dB SPL and at least 500 stimulations were performed on each test. Response reproducibility observed was of at least 50% correction between the A and B curves, and was regarded as passes in the newborn hearing screening, the presence of emissions by at least three frequency bands, considering the signal-to-noise ratio (S/N) greater than or equal to 3 dB at 1 kHz and 1.5 kHz and greater than or equal to 6 dB at 2 kHz, 3 kHz and 4 kHz.

### Result analysis

The data was analyzed by the Student's *t*-test with the GraphPad Instat software.

The signal-to-noise ratio of otoacoustic emissions was described as average, minimum, maximum and standard deviation.

To compare the signal-to-noise ratio of otoacoustic emissions between the right and left ears of the individual and between genders and ages, we employed the variance analysis (ANOVA), considering that the data followed a normal distribution.

The significance level was 95% with *p* < 0.05.

## RESULTS

We observed the presence of TEOAE in all newborns evaluated.

The averages of the signal-to-noise ratios (dB SPL) for the TEOAE of the preterm newborns, for different frequencies were: 4.39 (1 kHz); 10.39 (1.5 kHz); 11.77 (2 kHz); 12.54 (3 kHz) and 9.54 (4 kHz); and for full term newborns they were: 3.49 (1 kHz); 10.28 (1.5 kHz); 11.58 (2 kHz); 15.1 (3 kHz), and 12.65 (4 kHz).

**Figure 1:**
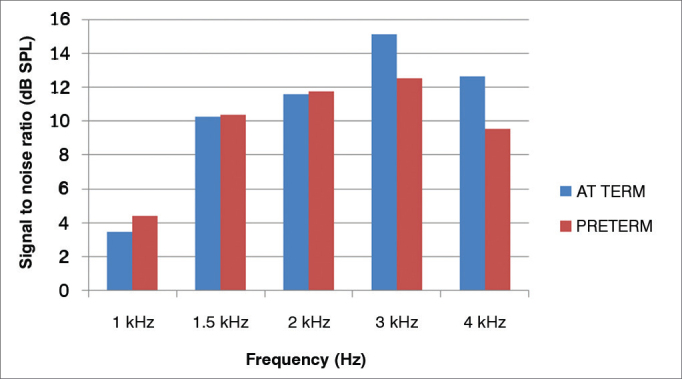
Mean levels of the signal-to-noise ratio (in dB SPL) in the sample studied, according to gestational age.

Figure 1 shows the signal-to-noise ratio mean values of the TEOAE in full term and preterm newborns.

For both groups, one can observe an increase of the amplitude response with increasing the frequency tested, except at 4 kHz.

In Table 1, Table 2, Table 3, Table 4, Table 5, Table 6 there is an exploratory analysis with the description of the TEOAE of our subjects.

**Table 1:** Description of the signal-noise ratio variable of TEOAEs (in dB SPL), for each frequency band, in both ears tested of full term newborns.

Ears FTNB (Frequency)	Signal-noise ratio						
	Mean	Maximum	Minimum	Median	CI < 95%	CI > 95%	*p*-value
RE (1,000 Hz)	2.89	10.78	-3.07	2.73	1.82	3.97	0.214
LE (1,000 Hz)	4.09	20.75	-2.64	2.66	2.48	5.69	
RE (1,500 Hz)	10.64	23.54	1.8	10.69	8.84	12.43	0.595
LE (1,500 Hz)	9.92	21.71	-2.14	7.63	7.89	11.95	
RE (2,000 Hz)	11.77	27.73	2.68	11.28	9.94	13.6	0.754
LE (2,000 Hz)	11.4	22.54	1.02	10.82	9.84	12.95	
RE (3,000 Hz)	13.47	25	4.64	12.31	11.76	15.17	0.01
LE (3,000 Hz)	16.74	27.54	4.28	16.81	14.9	18.57	
RE (4,000 Hz)	11.92	21.74	2.99	11.66	10.45	13.4	0.201
LE (4,000 Hz)	13.37	25.61	-2.05	13.5	11.65	15.1	

FTNB: Full Term Newborns; CI: Confidence Interval; RE: Right Ear; LE: Left Ear.

**Table 2:** Description of the signal-noise ratio variable of TEOAEs (in dB SPL), for each frequency band, in both ears tested of preterm newborns.

Ears PTNB (Frequency)	Signal-noise ratio						
	Mean	Maximum	Minimum	Median	CI < 95%	CI > 95%	*p*-value
RE (1,000 Hz)	5.21	15.98	-3.26	3.36	3.13	7.29	0.1747
LE (1,000 Hz)	3.47	13.08	-2.87	3.12	1.93	5.03	
RE (1,500 Hz)	10.65	27.34	0.13	9.38	8.01	13.3	0.7487
LE (1,500 Hz)	10.13	21.05	2.57	10.38	8.07	12.2	
RE (2,000 Hz)	11.2	23.8	2.55	10.79	8.43	13.96	0.5209
LE (2,000 Hz)	12.34	29.09	5.03	10.81	9.97	14.71	
RE (3,000 Hz)	11.41	28.41	4.16	10.62	9.27	13.55	0.1561
LE (3,000 Hz)	13.66	28.48	2.15	13.44	11.25	16.08	
RE (4,000 Hz)	9.13	21.45	-0.45	8.57	6.8	11.47	0.565
LE (4,000 Hz)	9.95	17.58	1.98	10.12	8.19	11.71	

PTNB: Preterm Newborns.

**Table 3:** Comparison of the signal-noise ratio variable of TEOAEs (in dB SPL), for each frequency band, found in full term and preterm babies, in both ears.

Ears FTNB (Frequency)	Signal-noise ratio						
	Mean	Maximum	Minimum	Median	CI < 95%	CI > 95%	*p*-value
FTNB (1,000 Hz)	3.49	20.75	-3.07	2.69	2.53	4.44	0.2824
PTNB (1,000 Hz)	4.39	15.98	-3.26	3.28	3.07	5.62	
FTNB (1,500 Hz)	10.28	23.54	-2.14	9.88	8.95	11.61	0.914
PTNB (1,500 Hz)	10.39	27.34	0.13	9.7	8.77	12.01	
FTNB (2,000 Hz)	11.58	27.73	1.02	11.27	10.41	12.76	0.857
PTNB (2,000 Hz)	11.77	29.09	2.55	10.8	10	13.53	
FTNB (3,000 Hz)	15.1	27.54	5.28	13.69	13.82	16.38	0.0139*
PTNB (3,000 Hz)	12.54	28.48	2.15	11.46	10.95	14.13	
FTNB(4,000 Hz)	12.65	25.61	-2.05	12.58	11.52	13.78	0.0008*
PTNB (4,000 Hz)	9.54	21.45	-4.45	9.73	8.13	10.96	

ATNB: At Term Newborns; PTNB: Preterm Newborns.

**Table 4:** Description of the signal-noise ratio variable of TEOAEs (in dB SPL), for full term newborns according to gender in the 1,000 to 4,000Hz frequency bands.

FTNB Gender (Frequency)	Signal-noise ratio						
	Mean	Maximum	Minimum	Median	CI < 95%	CI > 95%	*p*-value
M (1,000 Hz)	4.1	16.89	-3.07	3.45	2.02	6.18	0.4149
F (1,000 Hz)	3.24	20.75	-2.64	2.36	2.16	4.31	
M (1,500 Hz)	8.09	20.96	-2.14	6.95	5.52	10.66	0.0341*
F (1,500 Hz)	11.18	23.54	1.82	11.77	9.65	12.72	
M (2,000 Hz)	9.36	27.73	1.02	7.69	6.76	11.97	0.0146*
F (2,000 Hz)	12.5	23.94	3.21	12.07	11.26	13.75	
M (3,000 Hz)	14.83	27.54	4.28	14	12.2	17.46	0.2729
F (3,000 Hz)	15.21	26.41	4.92	13.32	13.72	16.71	
M (4,000 Hz)	11.98	23.66	-2.05	12.22	9.82	14.14	0.4472
F (4,000 Hz)	12.93	25.61	2.99	12.74	11.58	14.28	

FTNB: Full term newborns; M: Males; F: Females.

**Table 5:** Description of the signal-noise ratio variable of TEOAEs (in dB SPL), for preterm newborns according to gender in the 1,000 to 4,000Hz frequency bands.

PTNB Gender (Frequency)	Signal-noise ratio						
	Mean	Maximum	Minimum	Median	CI < 95%	CI > 95%	*p*-value
M (1,000 Hz)	5.83	14.87	-3.26	4.46	3.81	7.85	0.0223
F (1,000 Hz)	2.96	15.98	-2.38	2.56	1.44	4.49	
M (1,500 Hz)	13.33	27.34	0.13	12.9	10.58	16.08	0.0002*
F (1,500 Hz)	7.68	15.77	2.57	7.07	6.98	8.88	
M (2,000 Hz)	12.77	29.05	3.32	11.03	9.72	15.82	0.2728
F (2,000 Hz)	10.84	21.84	2.55	9.83	8.8	12.89	
M (3,000 Hz)	12.75	28.48	4.27	12.06	10.47	15.04	0.7939
F (3,000 Hz)	12.33	28.41	2.15	11.13	9.97	14.7	
M (4,000 Hz)	9.85	17.49	1.98	9.73	7.95	11.74	0.6831
F (4,000 Hz)	9.26	21.45	-0.45	9.42	7.06	11.47	

PTNB: Preterm newborn. M: Males; F: Females.

**Table 6:** Description of the signal-noise ratio variable of TEOAEs (in dB SPL), for full term and preterm newborns according to gender in the 1,000 to 4,000Hz frequency bands.

Gender (Frequency)	Signal-noise ratio						
	Mean	Maximum	Minimum	Median	CI < 95%	CI > 95%	*p*-value
M (1,000 Hz)	4.97	16.89	-3.26	3.84	16.89	6.38	0.022*
F (1,000 Hz)	3.15	20.75	-2.64	2.44	20.75	4.02	
M (1,500 Hz)	10.71	27.34	-2.14	11.02	27.34	12.68	0.5664
F (1,500 Hz)	10.1	23.54	1.82	8.99	23.54	11.27	
M (2,000 Hz)	11.07	29.05	1.02	9.61	29.05	13.06	0.369
F (2,000 Hz)	11.99	23.94	2.55	11.48	23.94	13.05	
M (3,000 Hz)	13.79	28.48	4.27	13.48	28.48	15.5	0.6166
F (3,000 Hz)	14.32	28.41	2.15	12.8	28.41	15.6	
M (4,000 Hz)	10.91	23.66	-2.05	11.01	23.66	12.33	0.3566
F (4,000 Hz)	11.79	25.61	-0.45	11.32	25.61	12.98	

M: Male; F: Female.

In the comparison between the ears of full term newborns we found a similar signal-to-noise ratio mean value in most frequencies, without statistical significance in the frequencies of 1 kHz; 1.5 kHz; 2 kHz and 4 kHz. There was only statistically significant difference in the 3 kHz frequency, with a better left-side mean value when compared to the right side (Table 1).

In comparing the ears of preterm newborns we found a similar signal-to-noise ratio mean value for the TEOAE test in all frequencies analyzed, without statistical significance (Table 2).

In comparing the signal-to-noise ratio of full term and preterm newborns, we found similar mean values in the frequencies of 1 kHz; 1.5 kHz and 2 kHz, with no statistically significant difference. There was a statistically significant difference in the frequencies of 3 kHz and 4 kHz, with a better mean value for the full term group than among the preterms (Table 3).

In comparing the signal-to-noise ratio of full term newborns, we found a statistically significant difference for the frequencies of 1.5 kHz and 2 kHz, with better mean values for females than for males in most frequencies analyzed (Table 4).

In comparing the signal-to-noise ratio of preterm newborns, we found a statistically significant difference for one frequency band only - 1.5 kHz, with better mean values for males than for females, with similar values in the remaining frequencies (Table 5).

When we studied the TEOAE signal-to-noise ratio (in dB SPL) in full term and preterm newborns of both genders, there was statistical significance only at 1 kHz and similar amplitudes in the remaining frequencies (Table 6.)

## DISCUSSION

The results showed the presence of TEOAE in 100% of full term and preterm infants. These results confirmed the findings by Peck^12^, who claimed to be possible to capture the movement mechanism of outer hair cells as of 25 weeks of gestation, when the peripheral auditory system is already formed.

The use of this test for hearing screening in newborns was effective, confirming reports from previous studies^13^, ^14^, which reported a greater response amplitude of evoked emissions than in their spontaneous counterparts, suggesting the use of this test as a hearing screening method as of 30 weeks of conceptional age.

In a study of cochlear function in neonatal ICU babies^15^, the authors reported that during the 30th to 40th week after conception, there was a 50-80% increase in otoacoustic emissions. The results suggest the clinical application of TEOAE to monitor cochlear function. For both groups there was an increase of the response amplitude with increasing the frequency tested, except at 4 kHz. Just like in another study^16^, which evaluated 526 newborns at full term and preterm, by TEOAE 48 h after birth, the authors reported better response amplitude in the higher frequency range, and the higher the post-conceptional age, the greater the response amplitude. They also observed better amplitudes for the right ear and in females; concluding that the otoacoustic emissions test may indicate peripheral auditory system maturation in newborns.

The present study also confirmed major differences in the higher frequencies, wherein maturation occurs later - as observed in another paper^9^ in which the authors reported that maturation does not occur uniformly at different frequency bands, showing an initial maturation at low frequencies, followed by the medium frequencies, and finally at the higher ones.

A similar study was carried out^11^ and no differences were found vis-à-vis TEOAE test response among infants born at full term and preterm, with and without auditory risk.

In this study, we observed that, in comparing the ears, full term and preterm subjects had similar responses in both ears, and statistical significance was found only in the frequency of 3,000 Hz in the full term group, with better values on the left side. We cannot infer a better response from any of the ears because the findings were very similar. In comparing genders, the females of the full term group had better mean values, with statistically significant differences for the frequencies of 1,500 Hz and 2,000 Hz. The preterm group had a statistically significant difference at the 1,500 Hz frequency only, with males having better amplitudes. When analyzing the full term and preterm groups, we found no differences between genders for any frequency. The same was not reported by other authors^14^ who recorded spontaneous and evoked otoacoustic emissions in 93 newborns at full term and preterm, and found a higher prevalence in females than in males and higher peaks in the right ear than in the left.

An evaluation of 582 full term neonates by means of otoacoustic emissions 48 hours after birth^17^ showed significant differences between genders and ears, with better amplitudes for females and the right ear.

These findings enable us to confirm that the TEOAE test provides important information on the peripheral auditory system of newborns at full term and preterm, and also because of the relative ease of recording it should be a method of choice in newborn hearing screening protocols for cochlear acuity assessment.

## CONCLUSION

The examination can be performed easily in newborns irrespective of gestational age.

From the result analysis we conclude that in comparing genders and ears, there was no difference in the signal-noise ratio between genders and the right and left ears vis-à-vis the TEOAE test in the groups of newborns at full term and preterm.

Improvements were seen in the signal-noise ratio with increasing the frequency bands evaluated.

With respect to gestational age, there were significant differences between the full term and preterm groups in the frequency bands of 3 and 4 kHz, with better responses for full term newborns.
